# Recovery of Cembratrien-Diols from Waste Tobacco (*Nicotiana tabacum* L.) Flowers by Microwave-Assisted Deep Eutectic Solvent Extraction: Optimization, Separation, and In Vitro Bioactivity

**DOI:** 10.3390/molecules29071563

**Published:** 2024-03-31

**Authors:** Tao Yu, Long Yang, Xianchao Shang, Shiquan Bian

**Affiliations:** 1 College of Plant Protection, Shandong Agricultural University, Tai’an 271018, China; 2 Anhui Provincial Key Laboratory of Rice Genetics and Breeding, Institute of Rice Research, Anhui Academy of Agricultural Sciences, Hefei 230031, China

**Keywords:** cembratrien-diols, natural deep eutectic solvent, response surface methodology, microwave-assisted extraction, antitumor activity

## Abstract

Deep eutectic solvents (DESs) are novel solvents with physicochemical properties similar to those of ionic liquids, and they have attracted extensive attention for the extraction of bioactive compounds from different plant materials in the context of green chemistry and sustainable development. In this study, seven DESs with different polarities were explored as green extraction solvents for cembratrien-diols (CBT-diols) from waste tobacco flowers. The best solvent, DES-3 (choline chloride: lactic acid (1:3)), which outperformed conventional solvents (methanol, ethanol, and ethyl acetate), was selected and further optimized for microwave-assisted DES extraction using the response surface methodology. The maximum yield of CBT-diols (6.23 ± 0.15 mg/g) was achieved using a microwave power of 425 W, microwave time of 32 min, solid/liquid ratio of 20 mg/mL, and microwave temperature of 40 °C. Additionally, the isolated CBT-diols exhibited strong antimicrobial activity against *Salmonella*, *Staphylococcus aureus*, *Escherichia coli*, *Bacillus subtilis*, and *Pseudomonas aeruginosa* and antitumor activity in the human liver cancer HepG2 and SMMC-7721 cell lines. This study highlights the feasibility of recovering CBT-diols from tobacco flower waste using DESs and provides opportunities for potential waste management using green technologies.

## 1. Introduction

Cembratriene-diols (CBT-diols) are cembranoid diterpenes with complex molecular structures [1] that were first extracted from burley tobacco leaves using traditional organic liquid–liquid extraction [2,3]. The parent skeleton is a fourteen-membered macrocycle composed of four isoprene units connected end-to-end [3]. CBT-diols are important aroma precursors that can be degraded to produce aroma components, such as solanone, solanifuran, and ketamine, which are key contributors to the aroma of tobacco [2]. Notably, CBT-diols have extremely high biological activity and are increasingly used in medicine and healthcare [4,5,6]. Therefore, the investigation of the biological activities of CBT-diols has recently become popular.

Tobacco (*Nicotiana tabacum* L.) is an important cash crop belonging to the *Solanaceae* family, which is distributed worldwide. Many chemical components are present in tobacco, and 5229 were confirmed [2]. Tobacco plants have well-developed glandular hairs and other secretory tissues in their epidermis, which produce many adhesive secretions during growth; CBT-diols are mainly present in these glandular hair secretions [2]. Additionally, recent research found that the content of CBT-diols in tobacco flowers is four to seven times higher than that in fresh tobacco leaves [2,7]. To date, traditional liquid–organic solvent extraction methods have been widely used for the effective extraction of CBT-diols from tobacco plants [2,8]. However, to minimize environmental pollution, new solvents are required to replace the organic solvents used for such extraction processes.

Natural deep eutectic solvents (DESs), also known as green eutectic solvents, are composed of natural compounds, for example, primary metabolites such as amino acids [9,10]. Owing to their unique physicochemical properties, DESs are considered a third liquid phase naturally present in organisms, independent of water and lipids [11,12]. Depending on the compounds used in their synthesis, DESs can be divided into five categories: ionic liquid, neutral, neutral acid, neutral base, and amino acid types [12,13]. Currently, choline chloride is the most widely used hydrogen-bond acceptors (HBAs) in DES systems and can form DESs with various hydrogen-bond donors (HBDs), such as urea, alcohols, carboxylic acids, and sugars [13,14]. The nontoxic, biodegradable, reusable, and green advantages of DESs make them popular for the extraction of active compounds [15,16]. For example, flavonoids [17] and phenolic acids [18] have been successfully extracted from different plant materials using DESs. Therefore, DESs are expected to replace traditional organic solvents and resolve many problems hindering the extraction of natural compounds.

In particular, microwave-assisted DES extraction can provide a safe, clean, and green extraction technique. Thus, the present study is the first to report on the extraction of CBT-diols from waste tobacco flowers using DESs in combination with microwaves. In particular, different DESs combined with microwave heating were used in this study to extract CBT-diols from tobacco flower waste, and the optimal conditions for the microwave-assisted extraction of CBT-diols from waste tobacco flowers were determined using single-factor experiments and a response surface methodology design. To demonstrate the biological activity of the CBT-diols extracted using DESs, their antimicrobial and antitumor activities were studied.

## 2. Results and Discussion

### 2.1. Evaluation of DES Extraction Efficiency

Recent studies confirmed that DESs have a high potential for the extraction of water-insoluble active compounds from various plant materials [16,19,20]. Therefore, in this study, seven DESs were tested as extractants for CBT-diols from WTFs. The compositions of the sustainable and environmentally friendly two-component DESs prepared in our work are listed in the Section 3.

**Figure 1:**
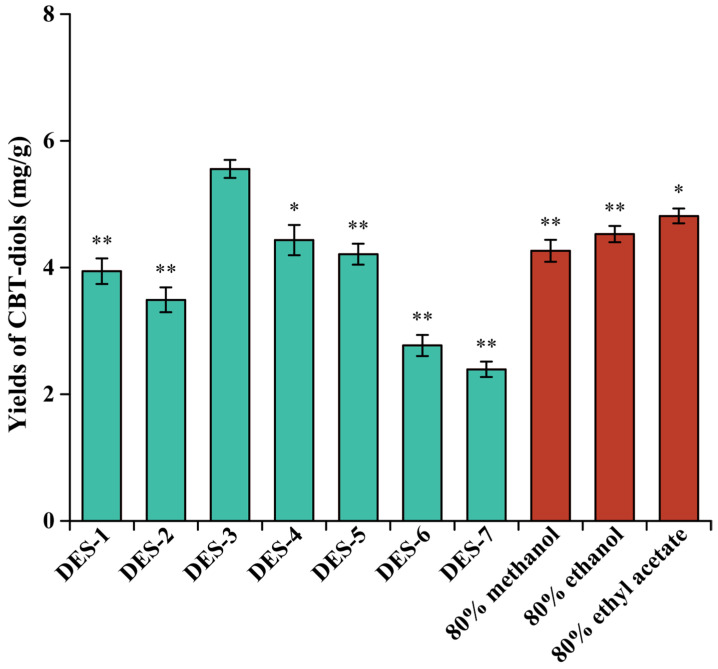
Comparison of CBT-diol extraction from WTFs using different DESs and three conventional organic solvents. Extraction parameters were as follows: microwave power of 300 W, microwave time of 20 min, microwave temperature of 40 °C, and solid/liquid ratio of 20 mg/mL. * and ** indicate that the extraction yields of CBT-diols from WTFs were significantly (*p* < 0.05) and extremely significantly (*p* < 0.01) different from that of DES-3.

Notably, the DES composition had a significant effect on the extraction efficiency (Figure 1). In particular, the DESs formed of HBA choline chloride exhibited the highest extraction efficiency, and the extraction yields of CBT-diols were 3.94 ± 0.20, 3.49 ± 0.20, 5.56 ± 0.14, 4.43 ± 0.24, and 4.21 ± 0.17 mg/g for DES-1 through DES-7, respectively. Crucially, DESs formed of carboxylic acids (lactic and citric acids in this study) as HBDs can efficiently damage and dissolve the cell walls of plants, thus improving the extraction efficiency of CBT-diols from WTFs [21]. Although DES-3 and DES-4 were composed of choline chloride and carboxylic acids, the high viscosity of DES-4 reduced mass transfer, which decreased the extraction yield of CBT-diols [13,22]. For DES-1, which was formed from polyols, the increase in the number of hydroxyl groups makes it easier for alcohols to form intramolecular hydrogen bonds with choline chloride, leading to an increase in the viscosity of the system and poor extraction efficiency of CBT-diols from WTFs [23]. In addition, to evaluate the DESs as a possible alternative to conventional solvents, a comparative analysis of the extraction efficiency was conducted using 80% methanol, 80% ethanol, and 80% ethyl acetate [13,24], indicating that the extraction effect of DES-3 was significantly better than that of traditional organic solvents. Overall, DES-3 (choline chloride and lactic acid at a molar ratio of 1:3) was selected as the optimum solvent for extracting CBT-diols from WTFs.

### 2.2. Optimization of DES Extraction Using Single-Factor Experiments

**Figure 2:**
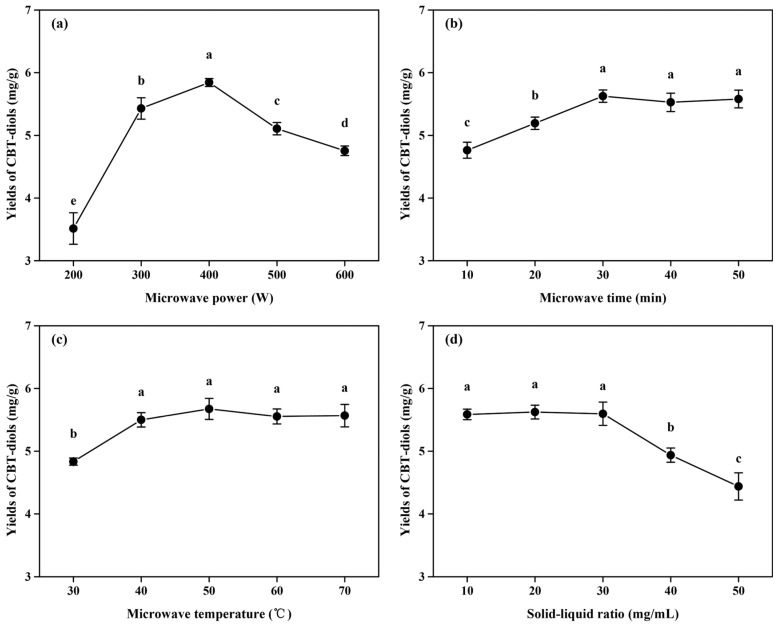
Single-factor experiments for optimizing the extraction efficiency of CBT-diols from WTFs. Effect of microwave power using DES-3 (**a**) with the following fixed parameters: microwave time of 20 min, microwave temperature of 40 °C, and solid/liquid ratio of 20 mg/mL; effect of microwave time using DES-3 (**b**) with the following fixed parameters: microwave power of 300 W, microwave temperature of 40 °C, and solid/liquid ratio of 20 mg/mL; effect of microwave temperature using DES-3 (**c**) with the following fixed parameters: microwave power of 300 W, microwave time of 20 min, and solid/liquid ratio of 20 mg/mL; effect of solid/liquid ratio using DES-3 (**d**) with the following fixed parameters: microwave power of 300 W, microwave time of 20 min, and microwave temperature of 40 °C. Different letters indicate significant differences at *p* < 0.05.

In addition to the type of extraction solvent, the extraction parameters (extraction power, time, temperature, etc.) have significant effects on the extraction yields of active substances from plant materials [25,26]. Therefore, the effects of microwave power, extraction time, temperature, and solid/liquid ratio on the yields of CBT-diols from WTFs using DES-3 were investigated (Figure 2).

#### 2.2.1. Effect of Microwave Power on the Yields of CBT-Diols from WTFs

As shown in Figure 2a, the yield of CBT-diols increased significantly as the microwave power was increased from 200 to 400 W. The maximum extraction yield was 5.84 ± 0.06 mg/g at microwave power of 400 W, possibly because the increase in microwave power caused a cavitation effect in cells, which enhanced cell wall breakage, reduced mass transfer resistance, and facilitated the extraction of CBT-diols [13,27]. However, the extraction efficiency of CBT-diols decreased significantly at microwave powers of 500 and 600 W, possibly because the excessive microwave power increased the solubility of impurities and damaged the structure of the CBT-diols, resulting in a decrease in extraction yield [28]. Thus, the optimal microwave power was set at 400 W for the RSM.

#### 2.2.2. Effect of Microwave Time on the Yields of CBT-Diols from WTFs

Generally, extraction time determines whether an active substance can be fully dissolved [13]. The effect of microwave irradiation time on the yield of CBT-diols is shown in Figure 2b. At short microwave times (10 or 20 min), the dissolution of CBT-diols from the WTFs was low, resulting in low yields of CBT-diols. When the reaction time was increased to 30 min, the yield reached a maximum of 5.63 ± 0.10 mg/g. Subsequently, the extraction yield showed no significant improvement with further extensions of the microwave time.

#### 2.2.3. Effect of Microwave Temperature on the Yields of CBT-Diols from WTFs

In the DES extraction process, microwave temperature is an important factor affecting extraction efficiency [9,29]. Temperature significantly affects the diffusion coefficient and viscosity of DESs, thereby affecting the extraction yield of CBT-diols [9,28]. As the microwave temperature increased, the yield of CBT-diols first increased and then stabilized (Figure 2c). Notably, the extraction yields significantly increased from 30 to 40 °C. This might be because the increase in microwave temperature reduced the viscosity of the DESs and increased the solubilities and diffusion coefficients of the CBT-diols [13,29]. At 40 °C, the highest yield was 5.50 ± 0.12 mg/g, and the yield remained basically unchanged above 40 °C.

#### 2.2.4. Effect of Solid/Liquid Ratio on the Yields of CBT-Diols from WTFs

The effects of different solid/liquid ratios on the extraction efficiency of CBT-diols were also investigated (Figure 2d). The extraction yields increased with decreasing solid/liquid ratio from 50 to 30 mg/mL. It is possible that, with the reduction in the amount of extraction material, the chance of contact between WTF cells and the DESs components increased, which is conducive to the dissolution of CBT-diols [13,30]. The yields of CBT-diols from WTFs were highest (5.60 ± 0.19 mg/g) at a solid/liquid ratio of 30 mg/mL. Therefore, 30 mg/mL was chosen for further optimization.

### 2.3. Optimization of DES Extraction Using the Response Surface Methodology

An RSM using BBD was applied to 29 experimental points based on the single-factor experiments [31,32]. The extraction yields of CBT-diols from WTFs are presented in Table 1, and the data analysis was performed using Design Expert 8.0 Trial. The following multiple quadratic regression model equation was obtained using the extraction yields of CBT-diols as the response values:

Yield = 6.03 + 0.1308*A* − 0.0508*B* + 0.0392*C* − 0.1458*D* − 0.0075*AB* + 0.0350*AC* − 0.2250*AD* − 0.1600*BC* − 0.0700*BD* + 0.0375*CD* − 0.7367*A*^2^ − 0.0542*B*^2^ − 0.2743*C*^2^ − 0.0268*D*^2^.

The results obtained using the regression equation are presented in Table 2. The *p*-value (<0.001) of the regression model indicates that it has extremely high significance (*p* < 0.01), and the *p* = 0.149 for lack of fit suggests that this is not significant (*p* > 0.05) (Shang et al., 2019). The *R*^2^ of the model was 0.9405, indicating a high correlation between the model and reality, and the fitting is good [33,34]. The coefficient of variation was 2.61% (<10%), and the signal-to-noise ratio was 11.7741 (>4), indicating that this model has high confidence and can be used to navigate the design space. The equation also showed a good fit with the experiments and could be used to analyze and predict the extraction yields of CBT-diols from WTFs using DESs. Further, the significance analysis of this equation indicated that the effects of primary terms *A* and *D*, interaction terms *AD* and *BC*, and secondary terms *A*^2^ and *C*^2^ on the extraction yields of CBT-diols reached a highly significant level, whereas the effects of the other terms were not significant. Based on the *F* value, the four factors affected the extraction yield in order of solid/liquid ratio > microwave power > microwave time > microwave temperature.

**Figure 3:**
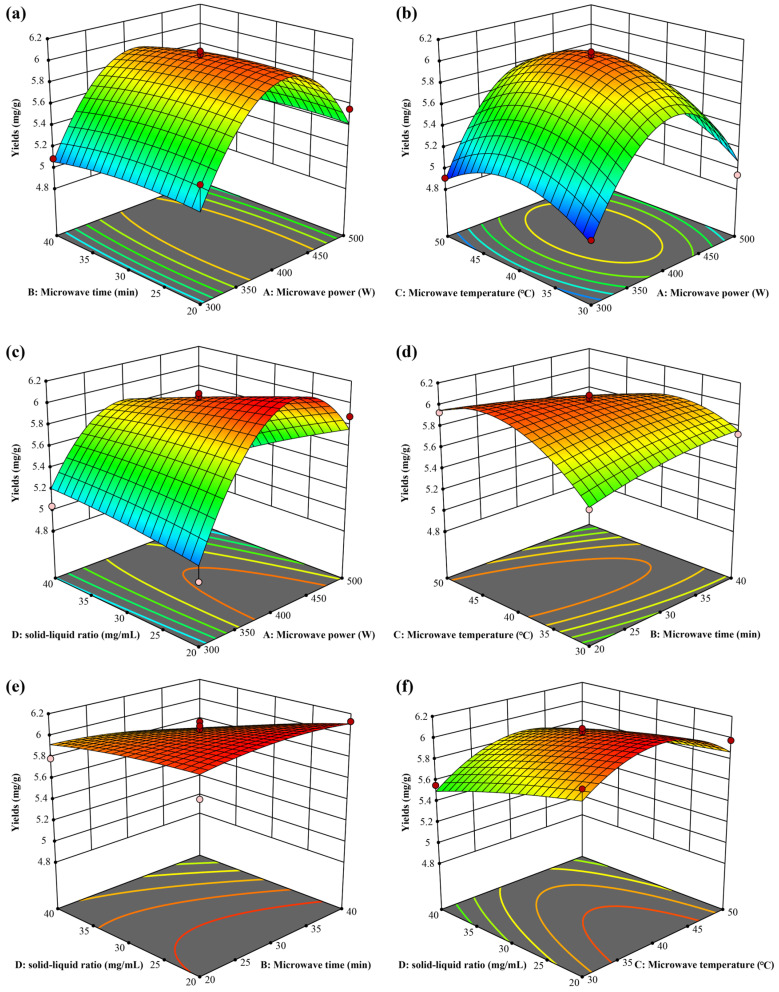
Three-dimensional RSM surfaces for optimization of DES extraction. Microwave power and microwave time (**a**); microwave power and microwave temperature (**b**); microwave power and solid/liquid ratio (**c**); microwave time and microwave temperature (**d**); microwave time and solid/liquid ratio (**e**); microwave temperature and solid/liquid ratio (**f**).

The model equation was used to plot the 3D response surfaces shown in Figure 3. As shown, the effect of microwave power on the extraction yield was significantly affected by the microwave power (Figure 3a–c). Further, the yield curve increased and then decreased, and hence, the optimal range of microwave power was determined to be 400–450 W. The combined effects of microwave time and temperature on extraction yield are shown in Figure 3d, even though they had no significant impact on the yield. However, the contour map shows that there was a significant interaction between microwave time and temperature. Thus, we concluded that the optimal microwave time was 30–35 min, and the optimal microwave temperature was 37–45 °C. By calculating the derivative of the model equation, we obtained the optimal conditions for extracting CBT-diols as follows: microwave power of 423.874 W, microwave time of 32.3123 min, microwave temperature of 39.5054 °C, and solid/liquid ratio of 20.2467 mg/mL. The theoretical extraction yield of CBT-diols was 6.18982 mg/g under optimal process conditions. For practicality, the extraction parameters were adjusted to a microwave power of 425 W, microwave time of 32 min, microwave temperature of 40 °C, and solid/liquid ratio of 20 mg/mL. The actual average extraction yield obtained from three replicate experiments was 6.23 ± 0.15 mg/g, which is close to the theoretical value.

### 2.4. Recovery of CBT-Diols from DES Extraction Systems

**Figure 4:**
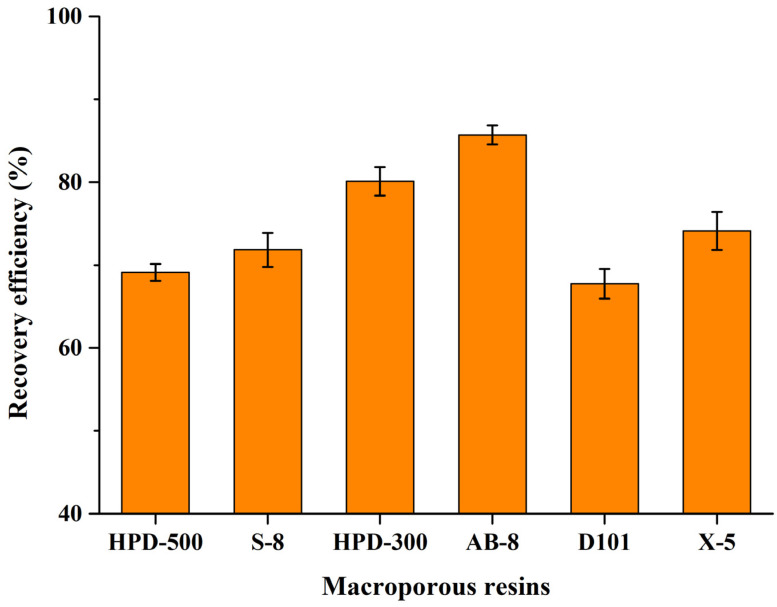
Recovery efficiency of six macroporous resins for CBT-diols.

To isolate CBT-diols from DES extracts and evaluate their biological activity, six different macroporous resins (HPD-500, S-8, HPD-300, AB-8, D101, and X-5) were tested. Analysis of the recovery efficiency (Figure 4) showed that the low-polarity macroporous resins (HPD-300 and AB-8) had the best adsorption capacities of all resins. In particular, AB-8 was the optimal macroporous resin for the adsorption and desorption of CBT-diols from DES extracts (recovery rate of 85.70% ± 1.14%). The recovery efficiencies could be influenced by the different properties of the six macroporous resins, such as polarity, particle size range, average pore size, and specific surface area. Therefore, the macroporous AB-8 resin was chosen for the isolation of CBT-diols from DES extracts.

### 2.5. In Vitro Bioactivity of CBT-Diols Extracted from WTFs Using DESs

#### 2.5.1. Antimicrobial Activity

The inhibitory activity of CBT-diols against fungi (*Botrytis cinerea* and *Phytophthora nicotianae*) was previously investigated [2]. To evaluate the antimicrobial activity of the isolated CBT-diols further, their antibacterial activities against *Salmonella*, *S. aureus*, *E. coli*, *P. aeruginosa*, and *B. subtilis* were determined. As shown in Table 3, CBT-diols from the WTFs and the positive control both exhibited inhibitory effects on all bacterial strains compared with the solvent control. Specifically, the antibacterial activity of the positive control was stronger than that of the CBT-diols. In addition, the isolated CBT-diols showed outstanding inhibition effects toward *S. aureus* (28.63 ± 0.55 mm) and *P. aeruginosa* (21.28 ± 0.63 mm). Thus, CBT-diols from WTFs extracted using DESs have good inhibitory effects on bacteria.

#### 2.5.2. Antitumor Activity

Many plant extracts were found to inhibit the growth of cancer cells dramatically [35,36,37]. For example, polysaccharides obtained from ginger (*Zingiber officinale*) directly inhibit the growth of the human colon cancer HCT 116 cell line, human lung adenocarcinoma H1975 cell line, and human cervical cancer HeLa cell line [38]. In addition, the total polyphenols from *Empetrum nigrum* significantly inhibit B 16F 10 melanoma cell proliferation and induce apoptosis in melanoma cells [39]. To determine the antitumor function of the isolated CBT-diols, we investigated the in vitro inhibitory activity of CBT-diols on the human liver cancer HepG2 and SMMC-7721 cell lines using the MTT assay.

**Figure 5:**
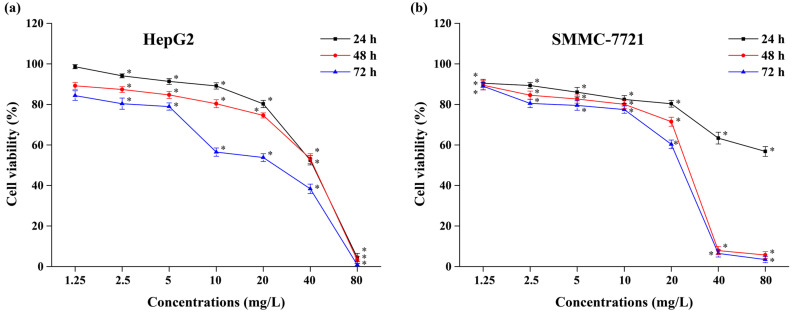
Effects of isolated CBT-diols on HepG2 (**a**) and SMMC-7721 (**b**) cell viability. * indicates that the cell viabilities in CBT-diols treatments were significantly (*p* < 0.05) different from the negative control treatments.

The antitumor activity of the isolated CBT-diols on HepG2 and SMMC-7721 cells is shown in Figure 5, revealing that it increased in a dose-dependent manner between 1.25 and 80 mg/L (Figure 5a,b). Primarily, the cell viability of HepG2 and SMMC-7721 decreased to 0.84% and 3.45%, respectively, at 72 h when using 80 mg/L. Compared with the negative control treatments, the inhibitory effect of the CBT-diols on HepG2 and SMMC-7721 cell proliferation in vitro was significant (*p* < 0.05). These results are consistent with those of previous studies. For example, cembratriene-4,6-diol extracted from cigarette smoke condensate inhibits cancer cell growth in vitro [40]. Therefore, we concluded that CBT-diols from WTFs extracted using DESs inhibited the self-proliferation of HepG2 and SMMC-7721 tumor cells in vitro.

## 3. Materials and Methods

### 3.1. Materials and Reagents

Fresh waste tobacco flowers (*N. tabacum* L., cultivated variety NC55) (WTFs) were collected from the Xinxing Experimental Station (Zhucheng City, Shandong Province, China; 36.048° N, 119.558° E). The WTFs were fully freeze-dried in a vacuum freeze-dryer (LGJ-10N/A, Beijing Yaxing Yike Technology Development Co., Ltd., Beijing, China) at −50 °C for 48 h. The dried WTFs were crushed into 40-mesh powder and then stored hermetically in a refrigerator at 4 °C before the extraction and quantification of the CBT-diols. Lactic acid, citric acid, glycerol, urea, d-(+)-glucose, tartaric acid, and choline chloride were obtained from the Aladdin Reagent Company (Shanghai, China). Acetonitrile for high-performance liquid chromatography (HPLC) analysis was purchased from Shanghai Macklin Biochemical Co., Ltd. (Shanghai, China). The other reagents and chemicals used in this study were of analytical grade and obtained from China National Medicines Co., Ltd. (Beijing, China).

### 3.2. Preparation and Characterization of DESs

All DESs used in this study were synthesized using the heating and mixing method described previously [13,41]. The components were added to capped flasks according to the molar ratios listed in Table 4, and a transparent homogeneous DES liquid was obtained by magnetic stirring in a water bath at 80 °C and 100 r/min for 3 h. Then, deionized water was added to the DES system to adjust the water content to 30% (*w*/*w*), and the samples were denoted DES-1 to DES-7. DESs diluted with water were used to extract CBT-diols from WTFs and to determine their viscosity and density.

### 3.3. Screening of DESs for the Microwave-Assisted Extraction of CBT-Diols from WTFs

To obtain the best DES system for the microwave-assisted extraction of CBT-diols from WTFs, the extraction capacities of the seven different DESs prepared in this study were evaluated by extracting WTF powder (100 mg) using 1 mL of DESs as the extraction solvent. The extraction processes were performed in triplicate at 40 °C for 20 min at a microwave power of 300 W. The yields of CBT-diols extracted from WTFs were used to compare the extraction efficiencies of the DESs with those of conventional solvents (80% methanol, 80% ethanol, and 80% ethyl acetate) [13,42].

### 3.4. Quantification of CBT-Diols Extracted from WTFs Using HPLC-UV Analysis

**Figure 6:**
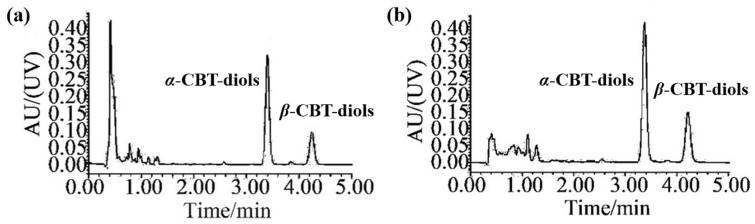
Chromatograms of α- and β-CBT-diols. Chromatograms of (**a**) CBT-diols in standard solutions and (**b**) CBT-diols in extracts from WTFs.

Quantitative analyses of the *α*-CBT-diols and *β*-CBT-diols isolated from WTFs were performed using ultra-HPLC (UPLC; H-CLASS, Waters, MA, USA). The chromatographic column was an Acquity UPLC BEH C18 column (1.7 μm, 50 mm × 2.1 mm). The mobile phase A and B were acetonitrile and pure water, respectively. The isogradient elution procedure was as follows: 0–8 min, 50% A and 50% B. The column temperature, injection volume, flow rate, and detection wavelength were 40 °C, 5 μL, 0.3 mL/min, and 200 nm, respectively. The chromatograms of *α*-CBT-diols and *β*-CBT-diols are shown in Figure 6. The calibration curve equation used for *α*-CBT-diols and *β*-CBT-diols determination were y = 20910x − 21414 (r^2^ = 0.9997, n = 8, with a linear range of 10–1000 μg/mL), and y = 15297x − 14940 (r^2^ = 0.9996, n = 8, with a linear range of 10–1000 μg/mL), respectively.

### 3.5. Single-Factor Optimization for the Extraction of CBT-Diols from WTFs

The single-factor experiments were designed and conducted to improve the efficiency of CBT-diols extraction from WTFs using DESs. Microwave power (200, 300, 400, 500, and 600 W), microwave temperature (30, 40, 50, 60, and 70 °C), microwave time (10, 20, 30, 40, and 50 min), and solid/liquid ratio (10, 20, 30, 40, and 50 mg/mL) were selected as the investigation factors. Based on the different parameters, CBT-diols were extracted from WTFs using DESs according to the methods described in Section 3.3. Single-factor experiments were performed using the extraction yields of CBT-diols as an optimization index.

### 3.6. Response Surface Methodology Optimization of the Extraction of CBT-Diols from WTFs

Based on the results of the previous single-factor experiments, Box–Behnken design (BBD) was applied to select suitable levels of each factor for response surface methodology (RSM) optimization using the central composite design principle. The four main influencing factors, microwave power, microwave temperature, solid/liquid ratio, and microwave time, were selected as independent variables. The final factor levels and coding values are presented in Table 5. After microwave extraction, the yield of CBT-diols isolated from the WTFs using DESs was used as the response value. The prediction model for the microwave-assisted extraction and correlation analysis of the responses and independent factors was developed using Design Expert Ver. 8.0 (Stat-Ease Inc., Minneapolis, MN, USA). Three-dimensional (3D) response surface plots were used to reveal the interactions between the various variables visually.

### 3.7. Recovery of CBT-Diols from DES Extracts Using Macroporous Resins

To recover the CBT-diols from the DES after extraction, six different macroporous resins (HPD-500, S-8, HPD-300, AB-8, D101, and X-5, Tianjin Yunkai Resin Technology Co., Ltd., Tianjin, China) were selected as separation and purification systems (Table 6). The pretreated macroporous resins (20 g) were added into a 100 mL syringe, and then 10 mL of the DES extract was added. Subsequently, the following elution procedure was used: thorough washing with 100 mL of deionized water (a flow rate of 2 mL/min) and elution with 50 mL of 50% ethyl acetate and 50 mL of 100% ethyl acetate (a flow rate of 1 mL/min). The ethyl acetate elution phases were entirely collected and dried using vacuum rotary evaporation. A crude extract of CBT-diols from the DES system was obtained and used for further analysis of biological activity. The recovery efficiency (%) of CBT-diols was calculated using the following equation:

Recovery efficiency (%) = (*C*_ea_ · *V*_ea_)/(*C*_e_ · *V*_e_),

where *C*_ea_ is the concentration of CBT-diols in the ethyl acetate elution phase, *V*_ea_ is the total volume of the ethyl acetate elution phase, *C*_e_ is the concentration of CBT-diols in the DES extract, and *V*_e_ is the volume of the DES extract.

### 3.8. Evaluation of In Vitro Bioactivity of CBT-Diols Extracted from WTFs Using DESs

#### 3.8.1. Evaluation of Antimicrobial Activity

The inhibitory effects of CBT-diols from WTFs by using DESs on *Salmonella* (CMCCBC2184B), *Staphylococcus aureus* (CMCCBC03068), *Bacillus subtilis* (CMCCB63501), *Escherichia coli* (CMCCBC00148), and *Pseudomonas aeruginosa* (CMCCBC0055B) were evaluated using filter paper diffusion methods, as described by Shang et al. [42]. These five bacteria were inoculated into Luria–Bertani (LB) agar medium, respectively, and cultured continuously at 37 °C for 12 h. A single bacterial colony was then selected and placed in 5 mL of liquid LB medium. The bacterial solution was incubated with oscillation until the optical density at 600 nm (OD_600_) was 0.6, and then it was stored at 4 °C. Under sterile conditions, a total of 100 μL of the bacterial suspension was uniformly coated on the surface of the LB agar medium. Sterile blank drug-sensitive tablets were soaked in CBT-diols dissolved in dimethylsulfoxide (DMSO, 150 μg/mL) for 30 s, dried slightly, and then attached to the surface of the medium. Drug-sensitive tablets containing penicillin–streptomycin solution (1000 U/mL) and DMSO solution were used as positive and solvent controls, respectively. All plates were incubated upside down at 37 °C for 24 h, and each treatment was repeated three times in parallel. Finally, the diameter of the inhibition zone was determined and used as an evaluation index for the antimicrobial activity of the CBT-diols.

#### 3.8.2. Evaluation of Antitumor Activity

The inhibitory effects of the isolated CBT-diols on the human liver cancer HepG2 and SMMC-7721 cell lines (Cell Bank of Chinese Academy of Sciences, Qingdao, China) in vitro were also determined using the colorimetric MTT assay (Shanghai Yuanxin Biotechnology Co., Ltd., Shanghai, China) [35,37]. The cell suspension was transferred into a culture bottle filled with complete culture medium, fully shaken, and then placed in a cell incubator (37 °C) containing 5% CO_2_ for incubation. DMSO solutions having different concentrations of CBT-diols (80, 40, 20, 10, 5, 2.5, and 1.25 mg/L) were used to detect the inhibition rate of the HepG2 and SMMC-7721 cells at 24, 48, and 72 h. Cancer cell culture media without CBT-diols were used as negative controls.

### 3.9. Statistical Analysis

Statistical analysis was conducted by analysis of variance (ANOVA) using SPSS 24.0 (Chicago, IL, USA). Means were compared by the Duncan test at a 95% confidence level. The experimental analysis was performed in triplicate, and data are represented as mean ± standard deviation.

## 4. Conclusions

The findings of this study demonstrate the feasibility of recovering CBT-diols from WTFs using DESs and the potential applications of CBT-diol-rich DES extracts. Among the tested DESs, DES-3, composed of choline chloride and lactic acid (molar ratio of 1:3), showed the highest CBT-diol extraction efficiency compared to the conventional organic solvents (80% methanol, 80% ethanol, and 80% ethyl acetate). The optimal conditions for microwave-assisted DES extraction were determined using RSM to be a microwave power of 425 W, microwave time of 32 min, microwave temperature of 40 °C, and solid/liquid ratio of 20 mg/mL. The yield of CBT-diols was 6.23 ± 0.15 mg/g under these conditions. The recovery of CBT-diols from the DES extraction systems was also investigated, and the macroporous AB-8 resin was selected as the most suitable separation resin. Furthermore, the antibacterial and antitumor activities of CBT-diols were evaluated in vitro. The isolated CBT-diols exhibited inhibitory effects against *Salmonella*, *S. aureus*, *E. coli*, *P. aeruginosa*, and *B. subtilis* also showed dose-dependent cytotoxic effects on HepG2 and SMMC-7721 human liver cancer cell lines. These results suggest that CBT-diols are promising antitumor agents and their mechanism of action warrants further investigation.

## Figures and Tables

**Table 1:** BBD and observed responses.

Run	Variables				Yields (mg/g)
	*A* (W)	*B* (min)	*C* (°C)	*D* (mg/mL)	
1	400	30	40	30	5.99
2	400	40	50	30	5.49
3	300	30	50	30	4.91
4	300	40	40	30	5.09
5	500	30	30	30	4.94
6	400	40	40	40	5.77
7	400	30	40	30	6.06
8	400	20	30	30	5.53
9	400	30	40	30	6.02
10	400	30	30	20	5.97
11	500	30	40	20	5.88
12	400	20	40	20	5.87
13	400	30	40	30	5.97
14	500	20	40	30	5.56
15	400	30	50	20	5.98
16	300	30	30	30	4.91
17	500	30	50	30	5.08
18	400	20	40	40	5.79
19	400	20	50	30	5.93
20	300	30	40	20	4.91
21	400	40	30	30	5.73
22	400	30	50	40	5.72
23	400	30	30	40	5.56
24	300	30	40	40	5.04
25	300	20	40	30	5.38
26	400	30	40	30	6.09
27	500	40	40	30	5.24
28	400	40	40	20	6.13
29	500	30	40	40	5.11

*A*, microwave power (W); *B*, microwave time (min); *C*, microwave temperature (°C); *D*, solid/liquid ratio (mg/mL).

**Table 2:** The ANOVA of this regression model for extraction yields of CBT-diols.

Source	Sum of Squares	Degrees of Freedom	Mean Square	*F* Value	*p*-Value
Model	4.67	14	0.3334	15.8	<0.0001
*A*	0.2054	1	0.2054	9.74	0.0075 **
*B*	0.031	1	0.031	1.47	0.2455
*C*	0.0184	1	0.0184	0.8725	0.3661
*D*	0.2552	1	0.2552	12.1	0.0037 **
*AB*	0.0002	1	0.0002	0.0107	0.9192
*AC*	0.0049	1	0.0049	0.2322	0.6373
*AD*	0.2025	1	0.2025	9.6	0.0079 **
*BC*	0.1024	1	0.1024	4.85	0.0448 *
*BD*	0.0196	1	0.0196	0.929	0.3515
*CD*	0.0056	1	0.0056	0.2666	0.6137
*A* ^2^	3.52	1	3.52	166.87	<0.0001 **
*B* ^2^	0.0191	1	0.0191	0.9048	0.3576
*C* ^2^	0.4879	1	0.4879	23.12	0.0003 **
*D* ^2^	0.0046	1	0.0046	0.22	0.6463
Residual	0.2954	14	0.0211		
Lack of Fit	0.2857	10	0.0286	11.76	0.149
Pure error	0.0097	4	0.0024		
Corr. total	4.96	28			

*A*, microwave power (W); *B*, microwave time (min); *C*, microwave temperature (°C); *D*, solid/liquid ratio (mg/mL). * *p* < 0.05 and ** *p* < 0.01.

**Table 3:** The diameter of inhibition zone of CBT-diols extracted from WTFs using DESs.

Samples	Diameter of Inhibition Zone (mm)				
	*Salmonella*	*S. aureus*	*E. coli*	*B. subtilis*	*P. aeruginosa*
CBT-diols	8.35 ± 0.57	28.63 ± 0.55	14.55 ± 0.69	15.02 ± 0.30	21.28 ± 0.63
Solvent control	-	-	-	-	-
Positive control	17.08 ± 0.77	32.17 ± 0.96	24.89 ± 0.52	24.37 ± 0.51	24.12 ± 0.65

- No inhibition zone.

**Table 4:** DES sample labels, compositions, molar ratios, densities, and viscosities.

Abbreviation	Composition		Molar Ratio	Density ^a^ (g/cm^3^)	Viscosity ^a^ (mPa/s)
	Component 1	Component 2			
DES-1	Choline chloride	Glycerol	1:2	1.18 ± 0.03	271.3 ± 1.8
DES-2	Choline chloride	Urea	1:2	1.08 ± 0.05	153.3 ± 0.9
DES-3	Choline chloride	Lactic acid	1:3	1.14 ± 0.07	105.5 ± 1.6
DES-4	Choline chloride	Citric acid	1:1	1.29 ± 0.04	128.9 ± 1.1
DES-5	Choline chloride	d-(+)-Glucose	3:2	1.28 ± 0.06	164.8 ± 1.4
DES-6	Urea	Glycerol	1:2	1.13 ± 0.03	143.6 ± 1.7
DES-7	Tartaric acid	d-(+)-Glucose	1:1	1.03 ± 0.05	129.6 ± 1.0

^a^ Density and viscosity were measured at 25 °C after dilution with water (30%).

**Table 5:** Independent variables and levels for BBD.

Variables	Symbols	Coded Levels		
		−1	0	1
Microwave power (W)	A	300	400	500
Microwave time (min)	B	20	30	40
Microwave temperature (°C)	C	30	40	50
Solid/liquid ratio (mg/mL)	D	20	30	40

**Table 6:** The characteristics of six macroporous resins for CBT-diols extracted from WTFs.

Macroporous Resins	Polarity	Specific Saturated Adsorption Capacity (mg/g)	Specific Surface Area (m^2^/g)	Average Aperture (nm)
HPD-500	High polarity	22.40 ± 0.0022	500–550	10–12
S-8	High polarity	21.57 ± 0.0028	100–120	28–30
HPD-300	Low polarity	41.16 ± 0.0075	800–870	5–5.5
AB-8	Low polarity	48.31 ± 0.0015	480–520	9–10
D101	Nonpolar	34.46 ± 0.0027	550–600	9–10
X-5	Nonpolar	32.19 ± 0.0070	650–700	9–10

## Data Availability

Data are contained within the article.
